# Harlequin Syndrome Caused by Multinodular Goitre

**DOI:** 10.7759/cureus.81298

**Published:** 2025-03-27

**Authors:** Andrew Strong, Justin Siew Yoong Tu, Sonya Kyi, Tissa Wijeratne

**Affiliations:** 1 Department of Neurology, Western Health, St Albans, AUS; 2 Department of Neurology, Western Health, La Trobe University, St Albans, AUS

**Keywords:** anxiety, dysautonomia, hemifacial, idiopathic, sympathetic fibers

## Abstract

Harlequin syndrome is a rare neurological disorder characterised by hemifacial flushing and sweating. We present a case of Harlequin syndrome with evidence of compression of the sympathetic trunk due to a non-toxic multinodular goitre. We review the neuroanatomy of this fascinating condition, particularly in relation to the more common and often sinister Horner’s syndrome, to convey a deeper understanding of the nuances of sympathetic facial innervation to the keen physician.

## Introduction

Harlequin syndrome is a rare neurological disorder characterised by hemifacial flushing and sweating. These episodes can be triggered by any combination of emotional responses, hot weather, and exercise [1]. Interestingly, whilst the prominent sympathetic symptoms may appear unilaterally, the culprit lesion is located contra-lateral to the side affected by the flushing. This is due to impaired sudomotor and vasomotor sympathetic output ipsilateral to the lesion, resulting in anhidrosis and lack of flushing. Diagnosis is often made clinically from suggestive symptoms and signs.

Anywhere along the three orders of sympathetic innervation to the face can be affected. Most isolated Harlequin syndrome is idiopathic in nature [2] but exclusion of underlying lesions should be investigated and may support the diagnosis. This is an important contrast to the rarely idiopathic Horner’s syndrome that can occur concurrently with Harlequin syndrome and should be one of the first things the astute physician looks for, after a thorough history [3]. In fact, when localising the site of a lesion, the concurrence of Horner’s is a useful neuroanatomical indicator.

We present a rare case of Harlequin syndrome secondary to a non-toxic multinodular goitre with tracheal deviation.

## Case presentation

A 49-year-old lady was referred to our neurology clinic with a four-year history of unilateral left-sided face flushing. Careful history taking revealed a background of a thyroid mass detected eight years before the onset of symptoms, which was found to be a non-toxic multinodular goitre. Her other past medical history was otherwise unremarkable.

**Figure 1:**
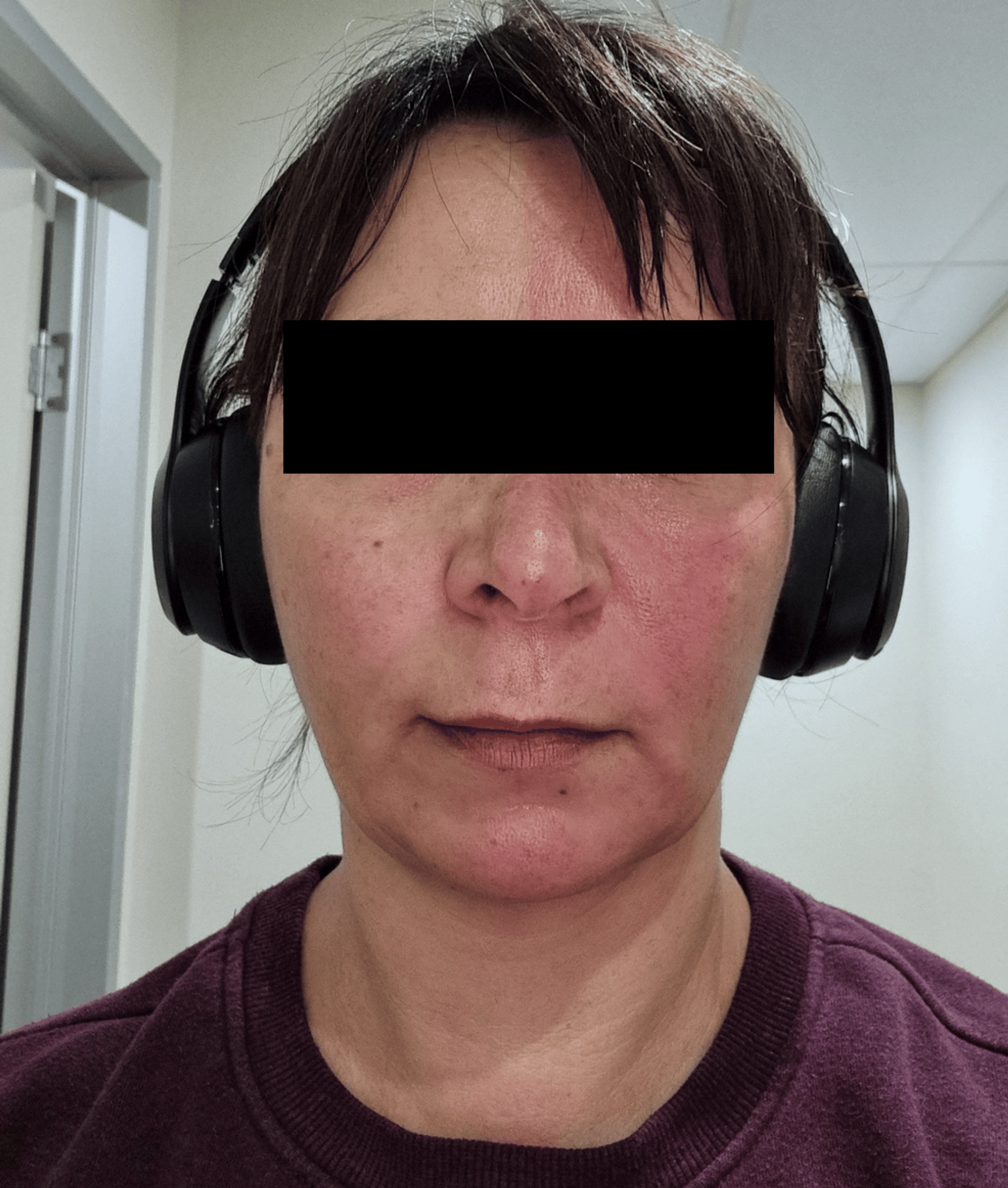
Patient post exercise with clearly demarcated left-sided facial flushing.

These symptoms occurred solely when exercising, and were absent at rest, in hot climates and in emotional states. Her symptoms were not associated with a headache, nor other autonomic features such as lacrimation or conjunctival injection. Photos taken after exercising demonstrate a clearly demarcated left facial flushing (Figure 1). On examination, a large right thyroid mass was palpable without other clinical features of hyperthyroidism or hypothyroidism. Her pupils were equal and reactive without ptosis. The remainder of her neurological examination was unremarkable.

**Figure 2:**
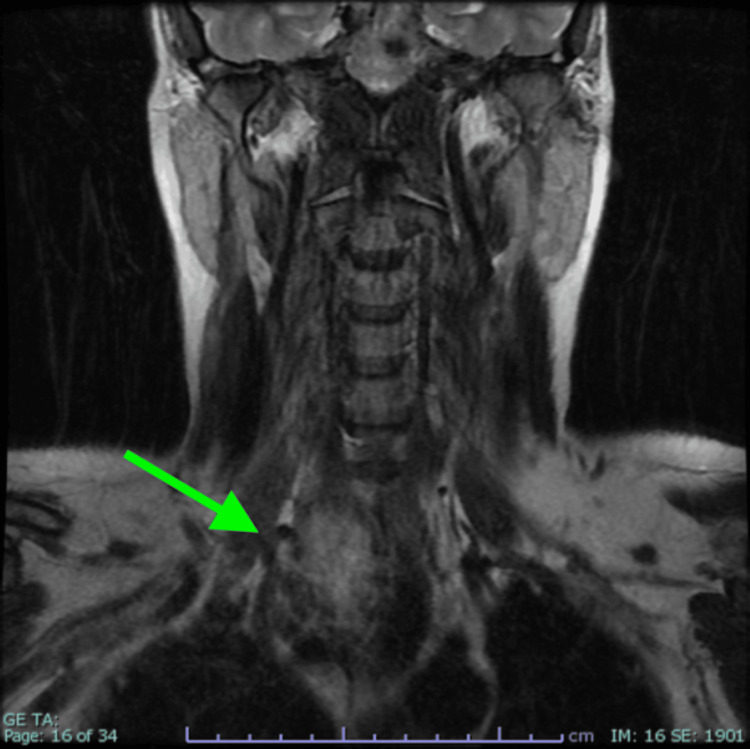
Initial MRI demonstrating a right sided thyroid mass (arrow) with compression and midline shift of the trachea.

An MRI of her neck (Figure 2) was conducted and discussed at our neuroradiology meeting, demonstrating an enlarged right hemi thyroid and tracheal deviation to the left. At this point, a diagnosis was made of left-sided Harlequin syndrome given this lady’s clearly defined left-sided facial flushing and sweating, with anhidrosis and absence of facial flushing on the right, and a neuroanatomically consistent lesion capable of right-sided compression of the sympathetic chain.

**Figure 3:**
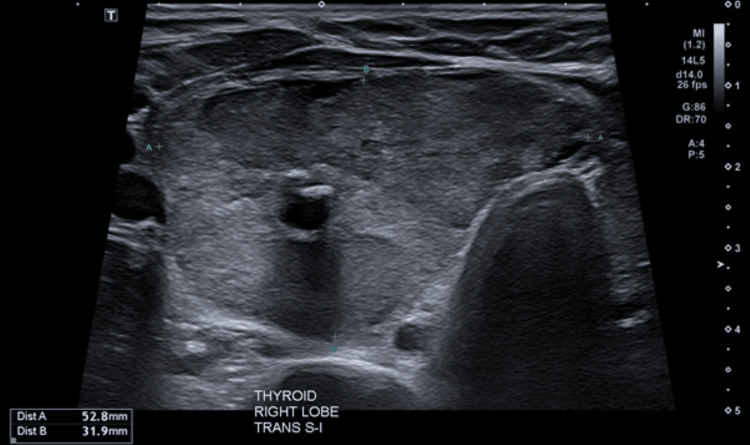
Ultrasound of the neck showing a dominant right-sided thyroid lesion measuring 62 x 52 x 32 mm.

Further investigation of the neck mass with ultrasound demonstrated several thyroid nodules bilaterally, with a dominant right lesion measuring 62 x 52 x 32 mm and a Thyroid Imaging Reporting and Data System (TI-RADS) score of 5 (Figure 3), alongside evidence of right-sided retrosternal extension. Fine needle aspirate of the dominant nodule at this time was indeterminate. She proceeded to have an uncomplicated total thyroidectomy and was commenced on thyroxine. Histology from her operation confirmed a diagnosis of a non-toxic multinodular goitre. 

**Figure 4:**
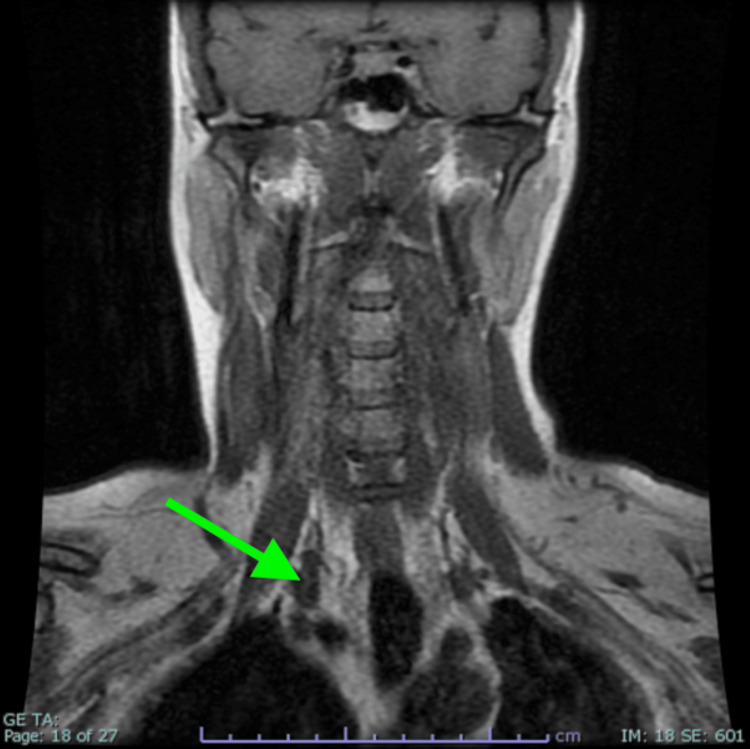
MRI post total thyroidectomy demonstrating resolution of tracheal midline shift, with an arrow identifying the surgical site of resection.

At our clinic review almost a year following her operation, our patient continues to experience unilateral face flushing with no subjective improvement, although she reports less fatigue. A repeat MRI (Figure 4) demonstrated a now midline trachea with no evidence of post-surgical complications or mass effect. Symptomatic treatment including propranolol and amitriptyline was offered to the patient at this time. However, this was declined by the patient as her symptoms only occur during exercise and do not affect her quality of life.

## Discussion

This case report highlights a rare instance of Harlequin syndrome secondary to a non-toxic multinodular goitre with tracheal deviation. The association between Harlequin syndrome and thyroid pathology underscores the importance of considering cervical and thoracic aetiologies in patients presenting with unilateral facial flushing and sweating. Despite surgical intervention with a total thyroidectomy, the patient remained symptomatic, suggesting a possible chronic alteration in the sympathetic pathways or compensatory mechanisms. Furthermore, our study provides a platform to discuss the interesting applied neuroanatomy of the oculosympathetic, vasomotor and sudomotor innervation of the face.

**Figure 5:**
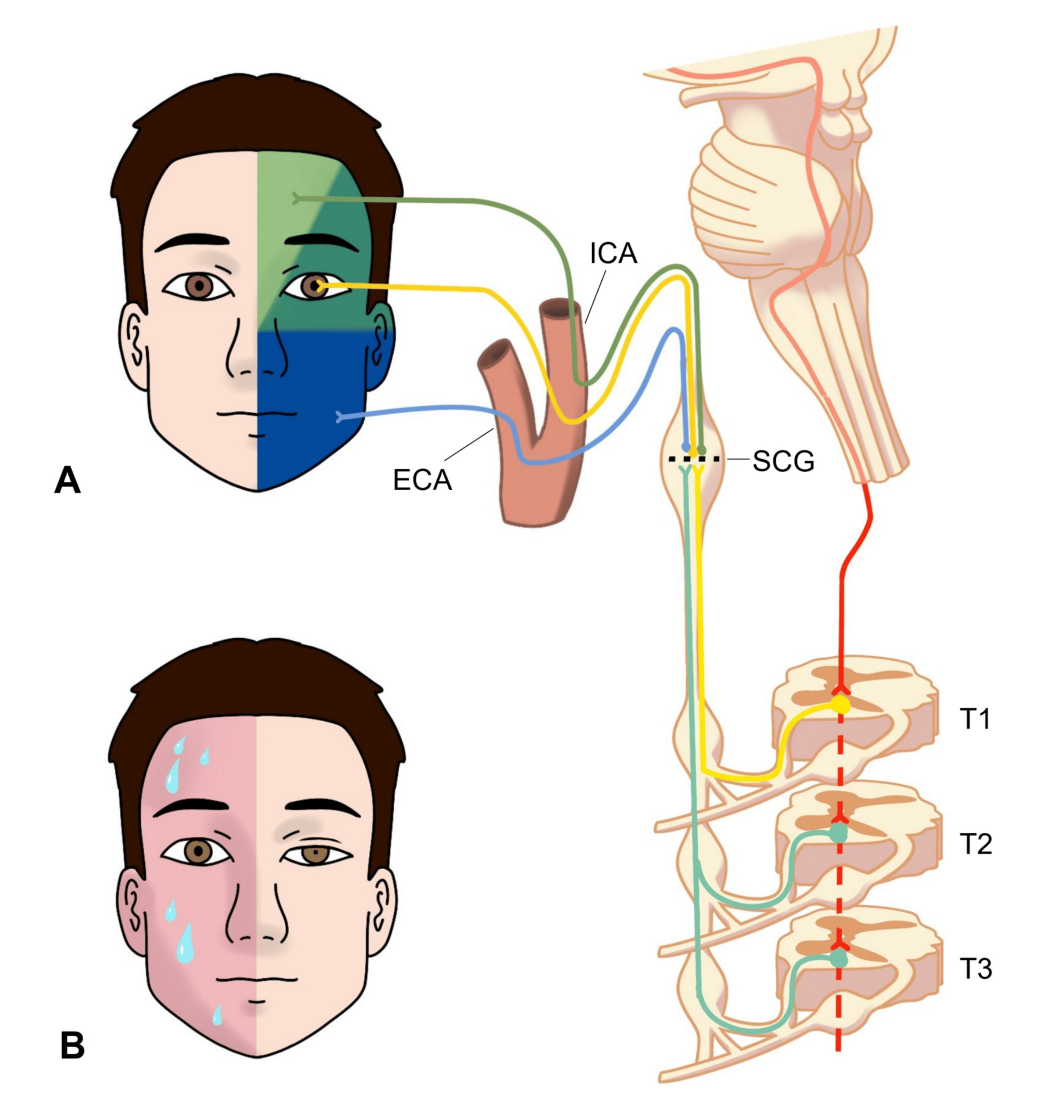
Sympathetic innervation of the face (A) First-order neurons (red) travel down from the hypothalamus, laterally in the brainstem and synapse in the intermediolateral spinal cord. Oculosympathetic preganglionic second-order neurons (light yellow) exit at the T1 level, with sudomotor and vasomotor neurons to the face (light green) exiting at the T2 and T3 level. All of these fibres synapse in the superior cervical ganglion (SCG). Postganglionic sudomotor and vasomotor fibres to the forehead (dark green) travel via the internal carotid plexus along the internal carotid artery (ICA). Sudomotor and vasomotor fibres to the rest of the face (blue) travel via the external carotid plexus along the external carotid artery (ECA). The cheek can be innervated by sudomotor and vasomotor fibres travelling on either the internal or external carotid. Oculosympathetic fibres (darker yellow) travel via the internal carotid plexus. (B) A face showing a left-sided Horner’s syndrome and right-sided Harlequin syndrome which, dependent on neuroanatomical location, could be caused by a lesion in the pathways shown in (A). Adapted from Wasner et al. [4] and Goadsby et al. [5] with permission.

The sympathetic innervation of the face originates in the hypothalamus. From the posterolateral hypothalamus, first-order fibres travel laterally through the brainstem to synapse in the intermediolateral column of the spinal cord (Figure 5). Second order (preganglionic) sudomotor and vasomotor fibres to the face exit from the spinal column at the T2-3 level, whereas oculosympathetic fibres exit the T1 level. Sudomotor and vasomotor fibres rejoin the oculosympathetic fibres as they ascend in the sympathetic chain and synapse in the superior cervical ganglion.

Finally, third-order (postganglionic) fibres once again diverge, with sudomotor and vasomotor fibres to the majority of the face travelling along the external carotid artery. However, it is a common misconception that all vasomotor and sudomotor fibres to the face travel with the external carotid artery. Sudomotor and vasomotor fibres to the medial forehead and brow area travel with oculosympathetic fibres along the internal carotid plexus [6]. Furthermore, fibres to the cheek may take either route with individual variation [7], as illustrated in the diagram. Sudomotor and vasomotor nerves reach the medial forehead via the ophthalmic nerve, diverging from oculosympathetic fibres [5]. Adding to the diagnostic complexity, affected sweat glands may also become reinnervated chronically by parasympathetic fibres [8].

Symptomatically, the concurrence of Horner’s syndrome and its preganglionic or postganglionic nature can help neurolocalise the causal pathology [4]. Lesions can occur anywhere along the three neuronal orders but most cases are idiopathic in nature [9]. For example, central brainstem infarcts and syrinxes in the spinal cord can lead to concurrent central Horner’s and Harlequin syndrome. The caveat should be noted that, while rare due to neuroanatomical proximity, central lesions have been reported that do differentially spare oculosympathetic or vasomotor/sudomotor fibres [8].

Pancoast tumours can invade upwards and compress the sympathetic chain, sometimes causing Harlequin syndrome prior to preganglionic Horner’s syndrome [10]. Similarly, thyroid nodules typically affect the sympathetic chain and may spare the sympathetic oculomotor system if it compresses at a point inferior to the point of T1 roots joining the sympathetic chain, such as in our patient. However, the inverse should not occur, as sudomotor and vasomotor fibres join oculomotor fibres as they ascend.

After the bifurcation of the carotid, causes such as internal carotid dissection can cause postganglionic Horner’s syndrome with medial forehead anhidrosis (and may also affect the cheek), sparing the rest of the face. Although typically transient, Harlequin syndrome can also occur with trigeminal autonomic cephalgias and migraine [11]. Finally, paratrigeminal oculosympathetic syndrome (also known as Raeder’s paratrigeminal syndrome) is caused by a lesion medial to the trigeminal nerve. It spares the sudomotor and vasomotor fibres to the forehead travelling via the ophthalmic nerve but affects oculosympathetic fibres following a different course [5].

Treatment may be as simple as counselling the patient on the normally benign nature of the condition. It can involve surgical removal of a compressive agent when present, but this may not resolve symptoms [12]. Instead, most interventions essentially serve to also block the sudomotor and vasomotor pathway on the side of facial flushing, contralateral to the lesion. This includes botulinum toxin injection [13], stellate ganglion block [14] and sympathectomy [15,16]. Propranolol and oxybutynin have also been reported to successfully treat the condition [17].

## Conclusions

This case report highlights a rare instance of Harlequin syndrome secondary to a non-toxic multinodular goitre with tracheal deviation. The association between Harlequin syndrome and thyroid pathology underscores the importance of considering cervical and thoracic aetiologies in patients presenting with unilateral facial flushing and sweating. There is a need for thorough clinical examination, extensive imaging and a broader understanding of the neuroanatomy involved in the sympathetic innervation to the face to accurately identify the potential causes in a patient presenting with Harlequin syndrome. This is particularly important when it presents with atypical features or in conjunction with other neurological signs. While the syndrome is generally benign and self-limiting, its dramatic presentation can cause significant distress to patients. Thus, proper diagnosis and patient education are crucial in managing expectations and providing reassurance.
